# Increased Accuracy of Genomic Prediction Using Preselected SNPs from GWAS with Imputed Whole-Genome Sequence Data in Pigs

**DOI:** 10.3390/ani13243871

**Published:** 2023-12-15

**Authors:** Yiyi Liu, Yuling Zhang, Fuchen Zhou, Zekai Yao, Yuexin Zhan, Zhenfei Fan, Xianglun Meng, Zebin Zhang, Langqing Liu, Jie Yang, Zhenfang Wu, Gengyuan Cai, Enqin Zheng

**Affiliations:** 1 National Engineering Research Center for Breeding Swine Industry, College of Animal Science, South China Agricultural University, Guangzhou 510642, China; yiyiliu0921@outlook.com (Y.L.); yulingzhang@stu.scau.edu.cn (Y.Z.); zfc17854225519@163.com (F.Z.); 14770011284@163.com (Z.Y.); zhanyx0923@outlook.com (Y.Z.); fanzhenfei3@gmail.com (Z.F.); a102818222105979@163.com (X.M.); zbzhang@scau.edu.cn (Z.Z.); langqing.liu@scau.edu.cn (L.L.); jieyang2012@hotmail.com (J.Y.); wzfemail@163.com (Z.W.); 2 Guangdong Provincial Key Laboratory of Agro-Animal Genomics and Molecular Breeding, South China Agricultural University, Guangzhou 510642, China; 3 Guangdong Zhongxin Breeding Technology Co., Ltd., Guangzhou 510642, China

**Keywords:** pigs, GS, accuracy, imputed WGS data, genome-wide association study, SNP preselection

## Abstract

**Simple Summary:**

By integrating prior biological information into genomic selection methods using appropriate models, it is possible to improve prediction accuracy for complex traits. In this context, we conducted a comparative assessment of two genomic prediction models, namely, genomic best linear unbiased prediction and genomic feature best linear unbiased prediction. The accuracy of these models in predicting the growth traits of backfat thickness and loin muscle area was evaluated. Our results revealed that the genomic feature best linear unbiased prediction model can effectively integrate prior information into the model, which is superior to the genomic best linear unbiased prediction model in some cases. These findings provide valuable ideas for enhancing the genomic prediction accuracy of growth traits in pigs.

**Abstract:**

Enhancing the accuracy of genomic prediction is a key goal in genomic selection (GS) research. Integrating prior biological information into GS methods using appropriate models can improve prediction accuracy for complex traits. Genome-wide association study (GWAS) is widely utilized to identify potential candidate loci associated with complex traits in livestock and poultry, offering essential genomic insights. In this study, a GWAS was conducted on 685 Duroc × Landrace × Yorkshire (DLY) pigs to extract significant single-nucleotide polymorphisms (SNPs) as genomic features. We compared two GS models, genomic best linear unbiased prediction (GBLUP) and genomic feature BLUP (GFBLUP), by using imputed whole-genome sequencing (WGS) data on 651 Yorkshire pigs. The results revealed that the GBLUP model achieved prediction accuracies of 0.499 for backfat thickness (BFT) and 0.423 for loin muscle area (LMA). By applying the GFBLUP model with GWAS-based SNP preselection, the average prediction accuracies for BFT and LMA traits reached 0.491 and 0.440, respectively. Specifically, the GFBLUP model displayed a 4.8% enhancement in predicting LMA compared to the GBLUP model. These findings suggest that, in certain scenarios, the GFBLUP model may offer superior genomic prediction accuracy when compared to the GBLUP model, underscoring the potential value of incorporating genomic features to refine GS models.

## 1. Introduction

Pork accounts for a large share of total global meat production, addressing the increasing demand for high-quality protein. Genetic factors play a predominant role among the various elements influencing the efficiency of the swine industry. It is essential to comprehend and optimize the genetic potential of pigs through genomic selection methods to enhance the efficiency and sustainability of pork production [1]. Genomic selection (GS), first proposed in 2001, is a statistical method for calculating the genomic estimated breeding value (GEBV) using high-density single-nucleotide polymorphisms (SNPs) across the whole genome [2]. This method hypothesizes that quantitative trait loci (QTL) for all target traits are in linkage disequilibrium (L) with at least one marker in the genome-wide high-density single-nucleotide polymorphism (SNP), so that the effect of each QTL can be reflected by the SNP [3]. The GEBV, calculated from individual genome data, outperforms the traditional estimated breeding value (EBV) based on pedigree records. This enhanced accuracy stems from GS, which leverages comprehensive genome-wide genetic marker information to more accurately depict the genetic relatedness among individuals [4]. Since GEBV can be independent of pedigree and phenotypic records, this facilitates selection early, which can significantly shorten the generation interval and increase the accuracy of predicted breeding values [5]. In pig breeding systems, the generation interval of pigs has been controlled for a short time and it is difficult to scale it down [6]. The GS of pigs is primarily based on improving the accuracy of GEBV to obtain additional genetic progression, and its accuracy depends largely on GS approaches.

The two major challenges of GS are the accuracy of GEBV and the cost of genotypes. SNP chip data have largely been the foundation for the adoption of GS throughout the past ten years. With the improvement of the reference genome sequence of livestock and the reduction in the cost of sequencing, whole-genome sequencing (WGS) data have become possible for GS. WGS data would include causal mutations that can find many QTL closely linked to targeting traits, which can greatly improve the accuracy of genomic prediction [7,8]. A study used simulation data to compare the accuracy of GEBV with a GBLUP model under low-density chip data (7.5K SNPs), high-density chip data (17K), and WGS data (335K), and found that the accuracy of GEBV based on WGS data was 4% higher than that based on chip data [9]. While the cost of WGS is falling rapidly, sequencing a large number of animals remains expensive. For most individuals, SNP high-density chips were used for genotyping, and the genetic variation in the whole genome obtained through genotype imputation would be cost-effective [10]. Currently, there are many livestock and poultry that perform GS based on imputed high-density genetic data or imputed sequence data, such as chickens [11,12], pigs [13], and cattle [14,15].

However, it was previously found that, compared to the GS based on chip data, using imputed WGS data for the accuracy of genomic prediction produced no advantage [9,15,16]. One of the important reasons for this is that prior information lacks consideration. At present, there are several ways to use prior biological information for GS. A common one is the use of QTL information, such as GS using QTL information in Holstein dairy cows, which improves accuracy by 4% [17]. Genome-wide association study (GWAS) is based on LD, identifying marker loci closely correlated with phenotypic variation by comparing the relationship between phenotypic differences in target traits across different individuals and the polymorphisms at genetic loci. Given the significance of GWAS in identifying candidate loci for complex traits in livestock and poultry, the selection of SNPs with significant effects on target traits based on GWAS results has become one of the most widely applied genomic-level priors [18,19]. Gebreyesus improved the accuracy of prediction through integrating prior information from GWAS results by 13~38% compared with the genomic best linear unbiased prediction (GBLUP) model in the study of milk fatty acid composition [20]. Subsequent studies also showed that significant SNPs found by GWAS using imputed WGS data can increase the accuracy of genomic prediction [8,10,21]. The genomic feature best linear unbiased prediction (GFBLUP) model, proposed by Edwards [22], can integrate biological prior information and treat markers that significantly impact traits as independent random effects within the model. This approach has been shown to enhance the accuracy of genomic prediction through the integration of prior information.

Duroc × Landrace × Yorkshire (DLY) hybrid pigs are the most widely bred pigs in the swine industry, as they have the advantages of fast growth and high feed utilization, providing consumers with the largest source of pork [23]. In this work, we adopt a research strategy of integrating prior information from GWAS results to evaluate the genomic prediction accuracy of the GBLUP and GFBLUP models using imputed WGS data. We collected important growth traits of pigs, backfat thickness (BFT) and loin muscle area (LMA). The objectives of this study were (i) to perform a GWAS on a total of 685 Duroc × Landrace × Yorkshire (DLY) pigs to extract significant SNPs as genomic prior information; (ii) to improve the accuracy of genomic prediction by integrating preselected SNPs from the GWAS into the prediction models using a population of 651 Yorkshire pigs.

## 2. Materials and Methods

### 2.1. Ethics Statement

All animals used in this study met the guidelines for the care and use of experimental animals established by the Ministry of Agriculture of China. The whole study was approved by the ethics committee of South China Agriculture University (SCAU, Guangzhou, China). The experimental animals were not anesthetized or euthanized in order to conduct this study.

### 2.2. Pig Population

The experimental animals in this study were two populations, the prior discovery population and the reference/validation population. A total of 685 DLY pigs (338 males and 347 females) were provided by Wens Foodstuffs Group Co., Ltd. (Yunfu, China) and served as the prior discovery population, born from May to August 2019. DLY pigs were bred from Duroc boars, including American Duroc (S21) pigs, Canadian Duroc (S22) pigs, and Taiwanese Duroc (S23) pigs, crossed with Landrace × Yorkshire sows. In total, 651 Yorkshire pigs provided by Guangdong Guangken Animal Husbandry Group Co., Ltd., born between July 2019 and October 2020, were used as the reference and validation populations. All pigs were raised with the same customized diet in human-controlled farm conditions and similar management conditions. The customized corn–soybean feed (free of probiotics and antibiotics) contained 16% crude protein, 3100 kJ of digestible energy, and 0.78% lysine. Water was available ad libitum. Feeding was completed when the body weight reached 100 ± 5 kg.

### 2.3. Phenotypic Data

According to the current effective standard, large-scale pig slaughtering experiments were carried out. Ear tissue was collected as follows: the pig’s ear was first cleaned with 75% alcohol. Then, a clear forfex was used to cut out a small fraction of ear tissue. The wound was then treated with tincture of iodine. The protocol for ear tissue collection was approved by the ethics committee of SCAU. The main growth traits of pigs were collected, that is, back fat thickness (BFT) and loin muscle area (LMA). BFT was collected with vernier caliper at rib 6–7 thoracic vertebra when pigs weighed 100  ±  5 kg, and the maximum height and width of the cross-sectional area of the longissimus dorsi muscle at the last rib were measured. LMA is calculated as follows:

$$LMA(cm^{2})=height\times width\times 0.7$$

After collecting phenotypic data, the original data underwent a correction process using the single-trait animal model in the BLUPF90 programs [24]. The model used for correction included fixed effects such as sex, age, and the first three principal components. The MVP package in R language was used to analyze the phenotypic distribution of the reference/validation population. The resulting corrected phenotypic values were used for all subsequent analyses.

### 2.4. Genotype Data

The genomic DNA was isolated and extracted from approximately 15~20 mg of ear tissue following the traditional phenol/chloroform method. The quality and quantity of the DNA samples were measured with a NanoDropTM 2000 (Thermo Fisher Scientific, Waltham, MA, USA) as previously described [25]. In total, 685 DLY pigs were genotyped using the GeneSeek Porcine 50K SNP Chip (Neogen, Lincoln, NE, USA). The original genotype data can be read using the GenomeStudio 2.0.5 software. The genotype quality control procedures were performed using PLINK v1.9 software [26] with the following criteria: (1) individual call rate  >  90%; (2) SNP call rate  >  90%; (3) minor allele frequency > 1%; (4) Hardy–Weinberg test *p*-value > 10^−6^; and (5) SNPs lacking informative data and those located on the sex chromosomes were removed. After quality control, a final dataset of 33,197 SNPs remained for DLY pigs. Similarly, 651 Yorkshire pigs were genotyped using the GeneSeek Porcine 80K SNP Chip, and for quality control. Due to the different chip types and densities used in these two populations, and in order to increase the density of genetic markers, we performed genotype imputation.

The imputation process of 50K SNP chip data and 80K SNP chip data to WGS genotypes was performed with the SWIM database. The SWIM pig haplotype reference panel, based on 2259 animals across 44 breeds, demonstrated robust performance in genotype imputation, achieving a concordance rate above 96% and an r2 of 0.85 [27]. Subsequently, the imputed WGS data were quality-controlled using the same standard as above. In the prior discovery population, 15,743,104 SNPs for DLY pigs remained after quality control. For the 651 Yorkshire pigs, 14,166,374 SNPs remained after quality control. The imputed WGS data were used for subsequent analysis.

### 2.5. Genetic Parameter Estimation

Heritability ($h^{2}$) was defined as the ratio of the additive genetic variance to phenotypic variance. Firstly, the genomic relationship matrix (GRM) was constructed to assess kinship among individuals. Subsequently, variance components were estimated using the restricted maximum likelihood (REML) algorithm via the GCTA 1.93.2 software [28].

### 2.6. Genome-Wide Association Study

For the two traits analyzed, the 685 DLY pigs were used to perform a GWAS and a genomic relationship matrix (GRM) based on all SNPs, which was constructed to account for population structure. The mixed linear model-based association analysis (MLMA) in the package GCTA [29] was used. The GWAS model was as follows:

y=1μ+Zg+bx+e

where y is the vector of corrected phenotypic value; μ is the overall mean, 1 is a vector of ones; g is the random effect, i.e., the accumulated effect of all SNPs, following a normal distribution $g∼N(0,G\sigma_{g}^{2})$, where G is the GRM which is built using imputed WGS data, captured genetic relatedness, $\sigma_{g}^{2}$ is the additive genetic variance, Z is the incidence matrix for g; b is the fixed effect of the candidate SNP to be tested for association, x is a vector of the SNPs’ genotype indicator variable coded as 0, 1, or 2; e is a vector of random residuals with $e∼N(0,I\sigma_{e}^{2})$, where $\sigma_{e}^{2}$ is the residual variance and I is an identity matrix. For ease of computation, $\sigma_{g}^{2}$ is estimated based on the null model and then fixed while testing for the association between each SNP and the trait.

### 2.7. SNP Preselection Based on the GWAS Results

After GWAS analysis, SNPs associated with BFT and LMA traits were categorized into different classes based on their *p*-values, specifically, those less than 0.05, 0.005, 0.0005, and 0.00005. Each trait resulted in four distinct sets of prior SNP information. Subsequently, these four sets of prior SNP information were individually intersected with the imputed WGS data of the 651 Yorkshire pigs. The common SNPs that are present in both the prior SNP information and the imputed WGS data of 651 Yorkshire pigs served as genomic features.

### 2.8. Genomic Prediction Models

The statistical methods used for predicting breeding value in this study included genomic best linear unbiased prediction (GBLUP) and genomic feature best linear unbiased prediction (GFBLUP) models. The genotype datasets for the GBLUP and GFBLUP models are imputed WGS data and preselected imputed WGS SNP data based on GWAS results. The GBLUP model is used to calculate GEBV as follows:

y=1μ+Zu+e

where y is the vector of the corrected phenotypic value; 1 is a vector of ones, μ is the overall mean; u is the vector of additive genetic values, and it is assumed that $u∼N(0,G\sigma_{u}^{2})$, where G is a relationship matrix built with the HIBLUP 1.1.0 software [30]; $\sigma_{u}^{2}$ represents corresponding additive genetic variance; Z is incidence matrices relating the additive genetic values to the phenotype value; e is the vector of random residual effect, and it is assumed that $e∼N(0,I\sigma_{e}^{2})$, where $\sigma_{e}^{2}$ is the residual variance and I is an identity matrix.

The GFBLUP model was an extended BLUP including two random genetic effects:

y=1μ+Zf+Zr+e

where y, 1, μ, and e are the same as in GBLUP, f is the vector of genetic effects captured by genomic features of target traits, following a normal distribution $f∼N(0,G_{f}\sigma_{f}^{2})$, r is the vector of genomic effects captured by the remaining genetic markers in the imputed WGS data that remove genomic features, following a normal distribution $r∼N(0,G_{r}\sigma_{r}^{2})$, and Z is an incidence matrix linking (f and r) to the phenotypic values. The $G_{f}$ and $G_{r}$ were constructed using only the genetic marker set defined by the genomic feature and the remaining set of markers; all of this was accomplished using the GCTA software. Since the computational resources of using two G matrices are too high, the two G matrices are combined into one G matrix to predict the GEBV:

$$G_{new}=\lambda G_{f}+(1-\lambda )G_{r}$$

where $\lambda =\frac{\sigma_{f}^{2}}{\sigma_{f}^{2}+\sigma_{r}^{2}}$, $\sigma_{f}^{2}$, and $\sigma_{r}^{2}$ are the genetic variances explained by $G_{f}$ and $G_{r}$ for BFT and LMA traits.

### 2.9. Prediction Accuracy

Cross-validation is a common method used to evaluate the performance of a model. In this study, five-fold cross-validation was performed to assess the prediction accuracy of the model, which was measured by the correlation coefficient between phenotype values and GEBV. Specifically, 651 Yorkshire individuals were randomly divided into 5 groups, with 4 groups serving as reference groups and the remaining group serving as the validation group. The genotypes and phenotype values of the reference groups were known and used to predict the model, while the genotypes of the validation group were known and the phenotype values were treated as missing values. This process was repeated for each group tested. The above steps were repeated 5 times, the prediction accuracy was reported as the average of these 25 results.

## 3. Results

### 3.1. Descriptive Statistics of Phenotypes and Heritability

Phenotypic statistics of BFT and LMA were performed in two populations. As Table 1 shows, the mean and standard deviation of BFT and LMA traits in DLY pigs were 11.49 ± 3.27 and 40.44 ± 7.39, and the coefficient of variation was 28.45% and 18.28%. It is implied that these two phenotypic variations are relatively stable in this population. Furthermore, the values (standard error) of the heritability estimates were 0.35 ± 0.08 and 0.34 ± 0.08 for BFT and LMA traits, both of which were medium heritability traits. Similarly, as shown in Table 2, the mean and standard deviation of BFT and LMA traits in Yorkshire pigs were 11.85 ± 2.43 and 40.34 ± 4.93, and the coefficient of variation was 20.54% and 12.21%. Data from Table 2 can be compared with the data in Table 1 which show that Yorkshire pigs’ heritability of BFT and LMA traits is 0.45, which is slightly higher than the heritability of BFT (0.35) and LMA (0.34) traits of DLY pigs. After phenotypic correction, BFT and LMA were roughly in line with normal distribution in the Yorkshire population (Figure S1), which could be used for GS.

### 3.2. SNP Preselection Based on the GWAS Results

The number of SNPs varies in GWAS results using imputed WGS data based on different levels of *p*-value (0.05, 0.005, 0.0005, 0.00005); see Table 3 for details. Based on the GWAS results, about 517.1K, 47.9K, 2.8K, and 171 significant SNPs were preselected according to different *p*-values for BFT traits in 651 Yorkshire pigs. In LMA traits, about 515.5K, 57.7K, 6.3K, and 341 significant SNPs were preselected according to different *p*-values as genomic features.

### 3.3. Genomic Prediction

**Figure 1:**
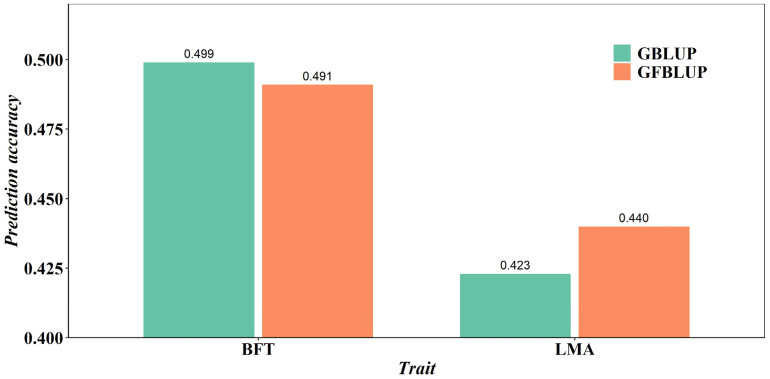
Accuracy of genomic predictions using GBLUP and GFBLUP models for BFT and LMA traits.

The prediction accuracy values of both GBLUP and GFBLUP models are shown in Table 4. The GBLUP model was run using the complete imputed WGS data, and the prediction accuracy values were 0.499 for the BFT trait and 0.423 for the LMA trait. Using SNP preselection based on the GWAS results with different *p*-value cutoffs, the prediction accuracy values of GFBLUP were 0.488, 0.487, 0.487, and 0.491 for the BFT trait and 0.440, 0.420, 0.417, and 0.423 for the LMA trait. The results showed that, for preselected SNPs from the GWAS results with the optimal *p*-value cutoffs (*p* < 0.05), the highest accuracy of the GFBLUP model was 0.440 for the LMA trait. This prediction accuracy value was still higher than that of the GBLUP model with the imputed WGS data. Using preselected SNPs from the GWAS results with the optimal *p*-value cutoffs, the accuracy of the GFBLUP model was lower than that of the GBLUP model for the BFT trait. Overall, the average prediction accuracy of BFT and LMA traits of the GFBLUP model reached the highest values at 0.491 and 0.440, respectively. The prediction accuracy of the GBLUP and GFBLUP models for BFT and LMA traits was visually compared, as depicted in Figure 1. In terms of BFT traits, there was no obvious trend for the accuracy of the GFBLUP model using different *p*-value cutoffs to preselect SNPs. However, for the LMA trait, the GFBLUP model improved prediction accuracy by 4.8% compared to the GBLUP model. In general, using the SNPs preselected from imputed WGS data based on GWAS results led to greater accuracy of genomic prediction for the LMA trait.

## 4. Discussion

In this study, an independent prior discovery population was specifically designed for GWAS analysis to select prior information. During the initial phases of GS research that integrated prior biological information, the availability of such prior information was relatively limited, often leading to a reliance on reference populations. Nevertheless, bias can be amplified when the prior discovery population and the reference population are the same or when GS relies solely on prior information [15,31,32]. For instance, in cattle research, utilizing the same prior discovery population for a GWAS as the reference population has been demonstrated to result in significant biases in GS [18]. In our research, in total, 685 DLY pigs were used as the prior discovery population for GWAS analysis, integrating completely independent GWAS results, and preselected significant SNPs were based on an independent population. In addition, a separate group of 651 Yorkshire pigs was used as the reference and validation populations to evaluate the prediction accuracy of the GS model. These two populations were relatively independent of each other (Figure S2).

Based on the GWAS results for BFT and LMA traits, significant SNPs were preselected for each trait and incorporated into the imputed WGS data for GS of the respective trait. Consequently, the number and specific sites of significant SNPs differed for each trait. The notable advantage of this study lies in the uniqueness of significant SNPs for each trait, potentially resulting in heightened prediction accuracy. However, it is worth noting that this study relies on prior GWAS information specific to individual traits. Therefore, in situations where a particular trait is not measured within the population or is challenging to obtain, the approach may not be universally applicable. Nonetheless, it is essential to acknowledge that this study relies on prior GWAS information tailored to individual traits. Thus, in cases where a specific trait is unmeasured within the prior discovery population or is challenging to acquire, the approach may not be universally applicable.

We explored the influence of different *p*-value cutoffs on preselected SNPs in genomic prediction, finding that the optimal *p*-value threshold significantly impacted prediction accuracy, though no definitive trend emerged across all cases. The preselected SNPs from the GWAS results were categorized based on different levels of *p*-values (0.05, 0.005, 0.0005, 0.00005). Similar categorization of preselected SNPs has also been utilized in previous studies [33]. While the categorization method may involve subjectivity, it is important to note that there is currently no strict standard in place. When the *p*-value is less than 0.05, the majority of potential genetic variants were selected. As the selection criteria become increasingly stringent, such as *p* < 0.00005, only a few hundred genetic variants remain as prior information. The outcomes of this study demonstrated that when using preselected SNPs from the GWAS results with the optimal *p*-value cutoffs (*p* < 0.05), the highest accuracy of GFBLUP was 0.440 for the LMA trait (Table 4). On the other hand, using preselected SNPs from the GWAS results with a *p*-value less than 0.00005 resulted in an accuracy of 0.491 for the BFT trait. It is worth noting that a study has speculated that as the number of preselected SNPs increases, the prediction accuracy of GS should first rise and then decline [15]. However, there was no distinct trend in the accuracy of GFBLUP using various *p*-value cutoffs for preselecting SNPs.

Incorporating prior biological information into GS has been shown to enhance the prediction accuracy for complex traits [34]. Presently, GS integrating prior biological information is widespread in the research focusing on important economic traits in cattle. In milk fatty acid-related traits, the prediction accuracy of GFBLUP integrated with GWAS results increased by an average of 23% in the Danish dairy cattle population and 13% in the Chinese population [20]. Regarding carcass traits in Hanwoo beef cattle, it was found that compared with the prediction accuracy using a 50K benchmark chip, using preselected SNPs from GWAS improved accuracy of prediction by 2.0% to 5.0% [32]. In a sheep study, the accuracy of GS for six meat traits and two wool traits was improved by integrating prior information based on GWAS with the foundation of the 50K chip [35]. A fundamental question revolves around the utilization of this prior information, especially in relation to selecting an appropriate GS model. In this study, we employed the GFBLUP model to integrate preselected significant SNPs. The widely used GBLUP model assumes that the influence of each genomic locus on the trait is uniform, thereby limiting its ability to integrate biological prior information. In contrast, the GFBLUP model overcomes this limitation by fitting two G matrices to assign different genetic weights to different classes of SNPs. Furthermore, the GFBLUP model allows for the fitting of more G matrices and further weighting of G matrices, by weighting multiple G matrices into a single G matrix to reduce computational resources, which can reduce computation time and memory. Consequently, this approach not only eases the memory demands for datasets featuring large sample sizes in the study but also enables the seamless integration of biological prior information into the modeling process. Overall, in terms of application scenarios, the GFBLUP model is more suitable for integrating prior information or other multi-omics data for prediction, as it can incorporate two or more random effects.

A comparison of the prediction accuracy of BFT and LMA traits by the GBLUP and GFBLUP models is depicted in Figure 1. For the GFBLUP model using preselected SNPs from the GWAS results with the different *p*-value cutoffs, the highest accuracy was 0.440 for the LMA trait and 0.491 for the BFT trait. The GBLUP model with complete imputed WGS data yielded accuracies of 0.423 for the LMA trait and 0.499 for the BFT trait. In the context of predicting the LMA trait in pigs, the GFBLUP model resulted in a notable 4.8% enhancement in genomic prediction accuracy compared to the GBLUP model. Previous research has also indicated that the GFBLUP model yielded a 38% enhancement in genomic prediction accuracy for bovine fatty acid traits [20]. Unfortunately, the prediction accuracy of the GFBLUP model did not meet expectations in predicting the BFT trait. This could be because the proportion of QTL in preselected genomic feature markers was very low, and using preselected SNPs would not be advantageous [36]. Similar results were also found in the Drosophila research [33] in terms of the starvation resistance trait. In a study of aquaculture species, it was observed that, compared with the prediction accuracy of the GBLUP model, the accuracy of the GFBLUP model integrating the GWAS results was reduced by an average of 6.2% in the prediction of disease resistance and growth traits [37]. If the proportion of QTL in preselected genomic feature markers was large, the GFBLUP model further increases its prediction accuracy compared to GBLUP with the complete WGS data [22]. With the same method, the differences in accuracy of genomic prediction between different traits may be due to the underlying genetic architecture [38]. Our future efforts will focus on using multi-omics data to select feature markers, ultimately improving genomic prediction accuracy.

## 5. Conclusions

Based on the GWAS results of 685 DLY pigs as prior information, we compared the GBLUP and GFBLUP models for accuracy of genomic prediction of two traits (BFT and LMA) by using the imputed WGS data of 651 Yorkshire pigs. Our results revealed that the average genomic prediction accuracy of the GFBLUP model for LMA trait was 4.8% higher than that of the GBLUP model. However, when predicting the BFT trait, both models exhibited comparable levels of prediction accuracy. This suggests that the GFBLUP model can effectively integrate prior information into the model, which is superior to the GBLUP model in some cases. Our findings provide valuable ideas for improving the genomic prediction accuracy of growth traits in pigs.

## Figures and Tables

**Table 1:** Descriptive statistics and heritability of BFT and LMA traits in DLY pigs.

Trait	Unit	Mean (±SD) ^3^	Min ^4^	Max ^5^	C.V./% ^6^	h^2^ (±SE) ^7^
BFT ^1^	mm	11.49 ± 3.27	5.50	25.58	25.48	0.35 ± 0.08
LMA ^2^	cm^2^	40.44 ± 7.39	20.25	64.63	18.28	0.34 ± 0.08

^1^ Back fat thickness (BFT). ^2^ Loin muscle area (LMA). ^3^ Standard deviations (SD). ^4^ Minimum (Min). ^5^ Maximum (Max). ^6^ Coefficient of variation (C.V.). ^7^ Heritability (standard error) value (h^2^ (±SE)).

**Table 2:** Descriptive statistics and heritability of BFT and LMA traits in Yorkshire pigs.

Trait	Unit	Mean (±SD) ^3^	Min ^4^	Max ^5^	C.V./% ^6^	h^2^ (±SE) ^7^
BFT ^1^	mm	11.85 ± 2.43	5.93	23.23	20.54	0.45 ± 0.06
LMA ^2^	cm^2^	40.34 ± 4.93	24.25	54.67	12.21	0.45 ± 0.07

^1^ Back fat thickness (BFT). ^2^ Loin muscle area (LMA). ^3^ Standard deviations (SD). ^4^ Minimum (Min). ^5^ Maximum (Max). ^6^ Coefficient of variation (C.V.). ^7^ Heritability (standard error) value (h^2^ (±SE)).

**Table 3:** Number of SNPs preselected based on the GWAS results with different *p*-value cutoffs.

*p*-Value	BFT		LMA	
	Number of SNPs in GWAS ^1^	Number of SNPs in Gf ^2^	Number of SNPs in GWAS ^1^	Number of SNPs in Gf ^2^
<0.05	731,061	517,173	720,230	515,502
<0.005	66,867	47,987	81,028	57,767
<0.0005	4399	2819	8020	6357
<0.00005	262	171	478	341

^1^ The number of SNPs divided according to *p*-value based on the GWAS results of DLY pigs (number of SNPs in GWAS). ^2^ The number of SNPs extracted as genomic features in Yorkshire pigs (number of SNPs in Gf).

**Table 4:** Prediction accuracies of the GBLUP model and the GFBLUP model with SNP preselection based on GWAS results.

Model	*p*-Value	Accuracy (Mean ± SE ^4^)	
		BFT	LMA
GBLUP ^1^	All ^3^	0.499 ± 0.016	0.423 ± 0.010
GFBLUP ^2^	<0.05	0.488 ± 0.017	0.440 ± 0.011
	<0.005	0.487 ± 0.017	0.420 ± 0.011
	<0.0005	0.487 ± 0.016	0.417 ± 0.010
	<0.00005	0.491 ± 0.016	0.423 ± 0.010

^1^ Genomic best linear unbiased prediction (GBLUP). ^2^ Genomic feature best linear unbiased prediction (GFBLUP). ^3^ All SNPs of imputed WGS data (All). ^4^ Standard error (SE).

## Data Availability

The genotype and phenotypic data analyzed in this study have not been made public at this time, as additional analytical studies will be conducted in the future. It can be obtained by contacting the corresponding author’s email address if reasonably requested.

## Supplementary Materials

The following supporting information can be downloaded at https://www.mdpi.com/article/10.3390/ani13243871/s1. Figure S1: Corrected phenotypic distribution of 651 Yorkshire pigs. (a) Phenotypic distribution of backfat thickness (BFT) trait and (b) Phenotypic distribution of loin muscle area (LMA) trait; Figure S2: Genetic structure of two populations (DLY and Yorkshire pigs), scatter plots of the first two principal components of genotype matrix for SNPs.
