# Supplementary material for: Increased Accuracy of Genomic Prediction Using Preselected SNPs from GWAS with Imputed Whole-Genome Sequence Data in Pigs
Source: Animals (Basel). 2023 Dec 15;13(24):3871. doi: 10.3390/ani13243871 (PMC10740755; doi:10.3390/ani13243871)
Supplement: Supplementary file 1 [file animals-13-03871-s001.zip › animals-2724435-supplementary.pdf]

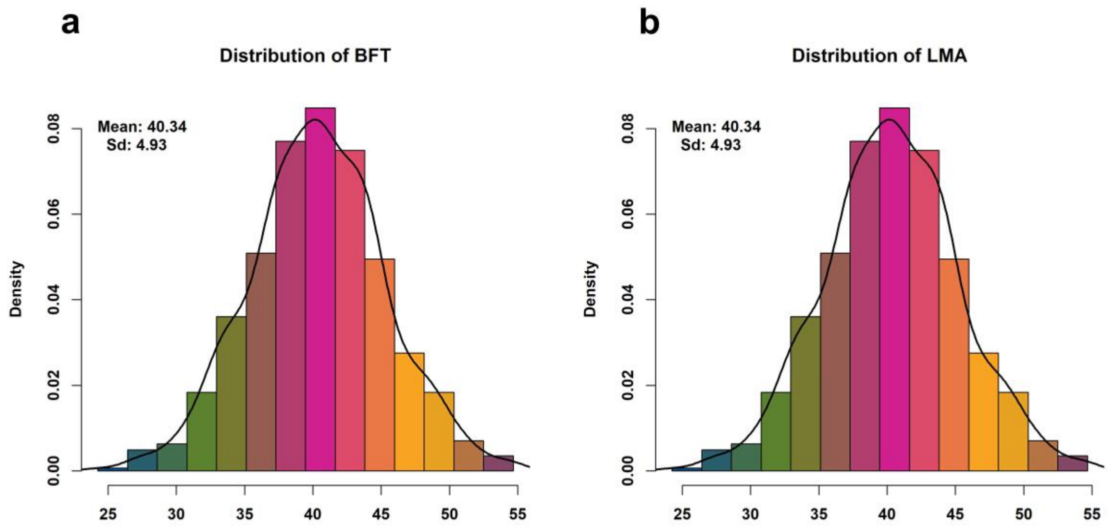

Figure S1: Corrected phenotypic distribution of 651 Yorkshire pigs. (a) Phenotypic distribution of backfat thickness (BFT) trait and (b) Phenotypic distribution of loin muscle area (LMA) trait;

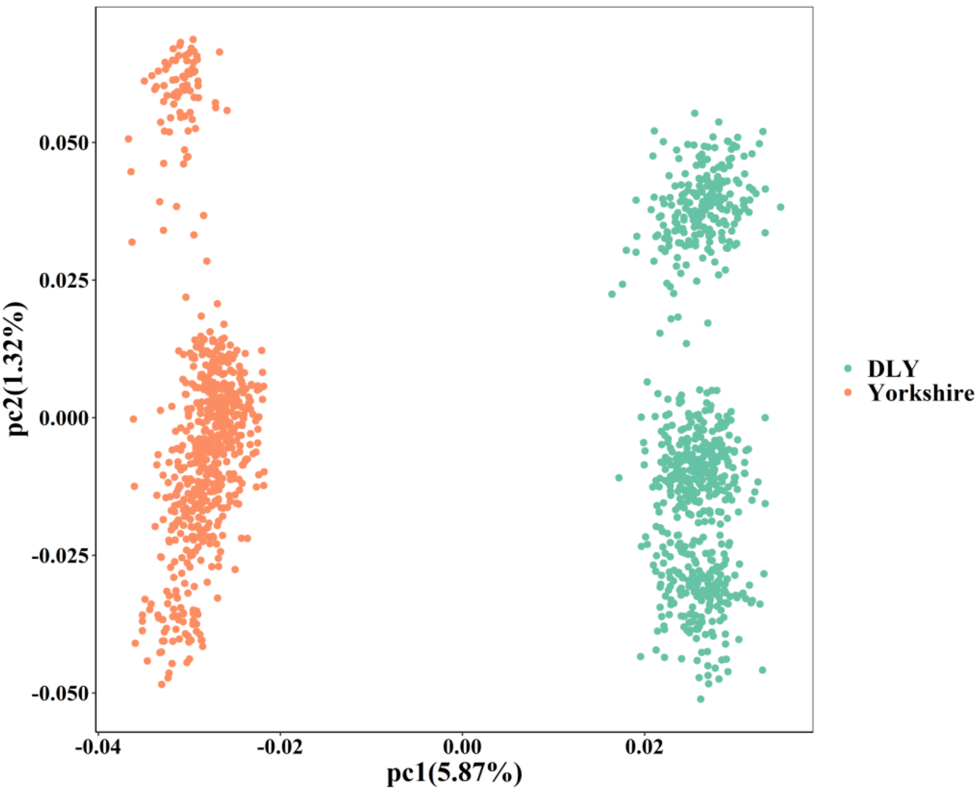

Figure S2: Genetic structure of two populations (DLY and Yorkshire pigs), scatter plots of the first two principal components of genotype matrix for SNPs
